# SamuROI, a Python-Based Software Tool for Visualization and Analysis of Dynamic Time Series Imaging at Multiple Spatial Scales

**DOI:** 10.3389/fninf.2017.00044

**Published:** 2017-06-29

**Authors:** Martin Rueckl, Stephen C. Lenzi, Laura Moreno-Velasquez, Daniel Parthier, Dietmar Schmitz, Sten Ruediger, Friedrich W. Johenning

**Affiliations:** ^1^Institute of Physics, Humboldt Universität BerlinBerlin, Germany; ^2^Neuroscience Research Center, Charité Universitätsmedizin BerlinBerlin, Germany; ^3^Berlin Institute of Health (BIH)Berlin, Germany; ^4^Einstein Center for NeuroscienceBerlin, Germany; ^5^Bernstein Center for Computational NeuroscienceBerlin, Germany; ^6^Cluster of Excellence ‘Neurocure’Berlin, Germany; ^7^DZNE-German Center for Neurodegenerative DiseaseBerlin, Germany

**Keywords:** calcium imaging, analysis software, Python programming, Open Source Software, microscopy, fluorescence

## Abstract

The measurement of activity *in vivo* and *in vitro* has shifted from electrical to optical methods. While the indicators for imaging activity have improved significantly over the last decade, tools for analysing optical data have not kept pace. Most available analysis tools are limited in their flexibility and applicability to datasets obtained at different spatial scales. Here, we present SamuROI (Structured analysis of multiple user-defined ROIs), an open source Python-based analysis environment for imaging data. SamuROI simplifies exploratory analysis and visualization of image series of fluorescence changes in complex structures over time and is readily applicable at different spatial scales. In this paper, we show the utility of SamuROI in Ca^2+^-imaging based applications at three spatial scales: the micro-scale (i.e., sub-cellular compartments including cell bodies, dendrites and spines); the meso-scale, (i.e., whole cell and population imaging with single-cell resolution); and the macro-scale (i.e., imaging of changes in bulk fluorescence in large brain areas, without cellular resolution). The software described here provides a graphical user interface for intuitive data exploration and region of interest (ROI) management that can be used interactively within Jupyter Notebook: a publicly available interactive Python platform that allows simple integration of our software with existing tools for automated ROI generation and post-processing, as well as custom analysis pipelines. SamuROI software, source code and installation instructions are publicly available on GitHub and documentation is available online. SamuROI reduces the energy barrier for manual exploration and semi-automated analysis of spatially complex Ca^2+^ imaging datasets, particularly when these have been acquired at different spatial scales.

## Introduction

Monitoring fluorescence changes of indicator molecules over time is one of the primary tools by which neuroscientists try to understand the function of neurons and neuronal networks. Small molecule indicators including Ca^2+^ and direct voltage sensors can be used to read out the spatiotemporal code of neuronal activity in a non-invasive way (Scanziani and Häusser, 2009) and are now routinely used in the study of brain activity at different spatial scales (Grienberger and Konnerth, 2012). As technological improvements allow imaging datasets to increase in their complexity (larger fields of view, longer permissible recording times, better temporal resolution, parallel use of different indicators at different wavelength), there is a growing need for tools that enable efficient data exploration. Furthermore, these tools must be applicable to datasets acquired at different spatial scales, because scientific questions increasingly require an understanding of processes at many scales sequentially or, if technically feasible, simultaneously.

Here, we would like to distinguish three spatial scales based on the existing terminology and the physical boundaries in conventional fluorescence microscopy with respect to resolution and field of view size: the subcellular or micro-scale, which includes subcellular structures like dendrites and spines (Jia et al., 2010; Kleindienst et al., 2011; Takahashi et al., 2012); the meso-scale, which comprises populations of individual cell bodies (Garaschuk et al., 2000; Stettler and Axel, 2009; Sofroniew et al., 2016); and the macro-scale, which is imaging of activity over several brain regions without cellular resolution (Conhaim et al., 2010; Busche et al., 2015). Datasets from each of these scales pose a different analytical challenge when extracting meaningful information about neuronal activity patterns from spatially defined regions of interest (ROIs).

In the last decade, we have seen major technical advances of genetically encoded Ca^2+^ indicators (GECIs) and the refinement of the multi cell bolus loading technique for *in vivo* Ca^2+^ imaging (Stosiek et al., 2003). These technical developments have led to a surge of Ca^2+^ imaging data at the meso-scale. Manual analysis of these datasets is labor intensive and can be prone to bias. This has driven the development of a wide variety of excellent tools that permit automated event detection and structure recognition for defining ROIs at the meso-scale (Junek et al., 2009; Mukamel et al., 2009; Tomek et al., 2013; Kaifosh, 2014; Hjorth et al., 2015; Pnevmatikakis et al., 2016).

While being tailor-made for meso-scale population Ca^2+^ imaging, these tools do not cover the requirements of other spatial scales. Batch processing and automation have enabled time-effective data analysis for large populations of cells, but similar advances have not been made in terms of data exploration and visualization of spatiotemporal structure. Both quality control (Harris et al., 2016) and manual identification of patterns in imaging data require intuitive and effective visualization. As far as we are aware, few analytical tools exist that provide users with an analysis environment that can be applied at different spatial scales. We hope that a user’s proficiency in handling data with SamuROI in a Python-based environment at one scale will greatly facilitate data analysis at other scales. This way, users should be able to reduce time and resources necessary to acquaint themselves with different analysis packages.

Furthermore, technological advances in microscopy have enabled longer observation periods in larger fields of view (Sofroniew et al., 2016), which together permit acquisition of spatiotemporally complex datasets. For example, it will soon be possible to routinely image thousands of cells at once (Harris et al., 2016). In this context, it becomes possible to address questions about spontaneous patterns of activity across multiple brain regions at different scales. The informational structure in spontaneous datasets is less predictable or manageable, and exploratory analysis is an essential step in making sense of the data. Data browsing tools are limited in this domain, and there is a need for tools that allow scientists to efficiently identify spatiotemporal structure within their data. We developed SamuROI to fill this niche: to provide a tool that enables analysis at multiple scales, and convenient data visualization for datasets with complex spatiotemporal structure.

SamuROI was designed for use on a standard desktop PC or laptop and focuses on intuitive data exploration and effective semi-automated ROI management. The built-in graphical user interface (GUI) displays data in the space, time and amplitude domains in a way that allows the user to easily connect fluorescence changes with their morphological location of origin and vice versa. This makes data inspection and manual curating of automated ROI generation easier and facilitates the rapid identification of data patterns during exploratory analysis. SamuROI has been designed to work alongside other software, and to link analytical tools developed for the micro-, meso-, and macro-scales. ROIs generated from other tools can be imported, and also modified manually. Datasets can be saved as hdf5 files in which both structural and dynamic information can be organized together. Hdf5 is also a suitable format for automated post-processing of the analyzed data using Python or other scripting languages. We take advantage of the interactive workflow provided by Jupyter Notebook, which allows seamless integration of the SamuROI GUI with custom pre- and post-processing analysis pipelines. This way, SamuROI bridges the gap between batch processing and data inspection while providing a versatile analysis environment for application in a range of imaging applications at different scales. SamuROI source code is publicly available on GitHub^1^ and licensed under the MIT license. Detailed installation instructions and usage documentation are also available online^2^. In this paper, we describe the software architecture and the general data processing workflow. We also provide examples of its application at the micro-, meso-, and macro-scale using Ca^2+^ imaging data obtained in acute slices.

## Materials and Methods

### Experimental Procedures

Experimental data used to demonstrate and evaluate the functionality of SamuROI was generated in accordance with the national and international guidelines. All procedures were approved by the local health authority and the local ethics committee (Landesamt für Gesundheit und Soziales, Berlin; animal license number T100/03).

Dendritic and spine calcium signals were obtained in layer 2 cells of the medial entorhinal cortex (MEC) in acute brain slices. Slices were prepared from juvenile Wistar rats (postnatal day 16 to 25) following the procedures as described in Beed et al. (2010). To provide optimal imaging conditions for small subcellular structures, we filled single cells with synthetic dyes. For dye filling, we either performed whole-cell patch clamp recordings or single cell electroporation for measurements where we did not want to interfere with the intracellular composition of the cell. The intracellular solution for filling patch clamp pipettes (3–6 MΩ) contained: 130 K-gluconate, 20 KCl, 10 HEPES, 4 MgATP, 0.3 NaGTP, and 10 phosphocreatine (in mM; pH: 7.3) and 30 μM Alexa 594 and 100 μM Oregon-Green BAPTA-6F (OGB6F). Electroporation pipettes were filled with 1 mM Oregon-Green BAPTA-1 (OGB1) and 150 μM Alexa 594 dissolved in ddH2O. The single 10 V electroporation pulse lasted 10 ms (Lang et al., 2006; Nevian and Helmchen, 2007).

For population Ca^2+^ imaging of neonatal spontaneous synchronous network events, we used the genetically encoded Ca^2+^ indicator (GECI) GCaMP6f. NEX-Cre mice (Goebbels et al., 2006) were crossed with Ai95 animals^3^ (Madisen et al., 2015) for constitutive GCaMP6f expression in excitatory cells only. Neonatal slices were cut horizontally for piriform cortex and sagittally for the parahippocampal formation at p0-10. We used the same ringer at all stages of preparation and recording. This solution consists of 125 mM NaCl, 25 NaHCO_3_, 10 mM glucose, 4 mM KCl, 1.25 mM NaH_2_PO^4^, 2 mM CaCl_2_ and 1 mM MgCl_2_, bubbled with carbogen (5% CO2 and 95% O2).

For all experiments, Ca^2+^ imaging was performed using a Yokogawa CSU-22 spinning disk microscope at 5000 rpm. The spinning disk confocal permitted the generation of a large field of view time series at a high acquisition rate. A 488 nm LASER was focused onto the field of view using a 4×, 40×, or 60× objective. Emission light was filtered using a 515 ± 15 nm band-pass filter. Fluorescence was detected using an Andor Ixon DU-897D back-illuminated CCD, with a pixel size of 16 μm. Andor iQ software was used for data acquisition. In order to prevent photo bleaching while producing the clearest images possible, we minimized the illumination power.

### Software Architecture

#### Requirements for Running SamuROI

In order to provide maximum backward compatibility, SamuROI is completely developed and tested using Python version 2.7. It should be possible to use SamuROI with Python versions 3.x but we have not tested this specifically. The efficient and effective use of SamuROI depends on four freely available python libraries:

- Numpy and scipy are libraries for dealing with numerical data in Python. They provide numerical routines for array manipulation and are capable of handling large datasets.
- PyQt, the bindings for the C++ widget library Qt is used for putting together windows, widgets, and other GUI elements.
- Matplotlib, a plotting library which allows plots to be embedded in PyQt widgets.

All four modules are widely used, under active development and have been rigorously tested and validated by the open source community. Throughout the development of SamuROI, we tried to build on top of the most recent versions of those projects. The source code of SamuROI is publicly available on GitHub^4^ (see README.md for installation instructions). SamuROI is licensed under the MIT license. The documentation of SamuROI is automatically built via Sphinx and available online^5^. Unit tests and a continuous integration pipeline of new releases are currently not available. Contributions in the form of bug reports, pull requests and proposed improvements are highly appreciated.

#### Basic Software Design of SamuROI

When designing SamuROI, a key objective was to provide easy extensions for custom functionality such as data pre- and post-processing, visualization and data curation. For this, toggling between the GUI and python code is central. We therefore encourage running SamuROI from a Jupyter Notebook, which provides easy access to all aspects of data management.

**FIGURE 1:**
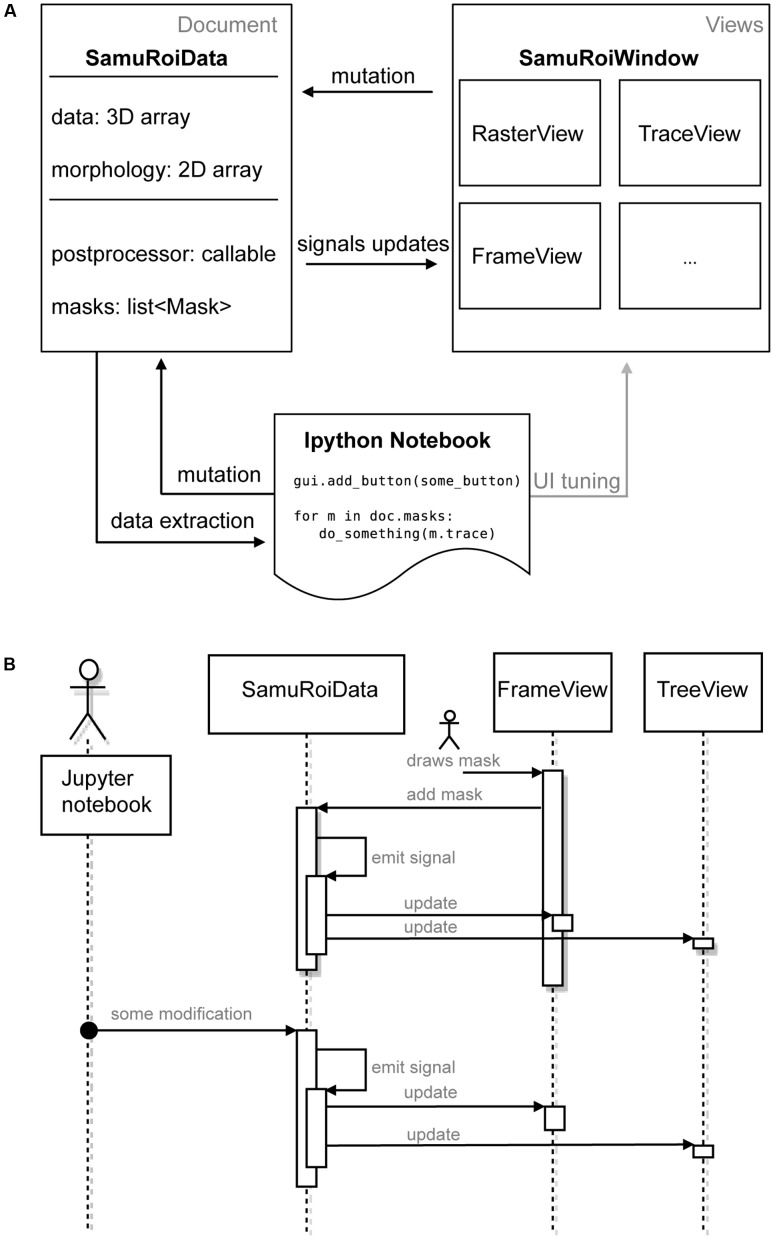
Software Architecture. **(A)** Diagram depicting the components of SamuROI in the document view pattern. SamuROI is split up into a data or document class (SamuRoiData) and multiple widgets which display the data (views). Communication between those classes takes place via callbacks (termed mutation, signals updates and data extraction in the diagram). This allows the user to update both, document and view by an interactive shell (here Ipython, bottom) without having to worry about keeping document and view in sync. **(B)** Flowchart to illustrate the process of signaling between widgets, data and interactive shell. User interactions are symbolized with the stickman.

We did not want the user to have to keep modifications via the GUI and the Jupyter Notebook in sync. For coordinating the different levels of interaction with the data via widgets in the GUI and the Jupyter Notebook, we implemented a strict separation of data and its presentation. Technically speaking, we used the “document-view” also known as “model view (controller)” design pattern (Gamma et al., 2015). In document view, data (Document in **Figure 1A**, i.e., the SamuROIData class), and its presentation to the user (Views in **Figure 1A**, i.e., the GUI and its widgets) do not depend on one another. For communication between these parts we use a signal slot pattern (as in Qt, sometimes also called “Observer pattern”) (Gamma et al., 2015) (**Figure 1A**). As data is mutated, the data object calls all slots of the respective signal, i.e., it informs all ‘listeners’ that some aspect of the data has changed.

The right hand side of **Figure 1A** represents a set of different views. All views ‘listen’ to signals of the data that are of relevance for their visualized content upon application start up, which means they are slots of the signal. If the data changes, signals will be emitted to all slots and, consequently, all listening views will be notified and will modify their presentation to the user accordingly.

The SamuROIData class on the left hand side of **Figure 1A** holds all relevant data and provides functionality for mutating and extracting subsets of data (**Figure 1A**, left). The most important data members of this class are:

- the 3D numpy array containing the video data
- the 2D numpy array containing the overlay mask
- multiple python containers holding user defined ROI objects

The extensive use of python properties within the SamuROIData class allows mutations of the data to be intercepted and the respective signals to be triggered. For the full API of the SamuROIData class and the mask sets, the reader is referred to the online documentation, especially the examples section. The signals provided by the SamuROIData class are trivially implemented as lists of python functions where the arguments of the signal invocation get perfectly forwarded to all functions contained in the list:

class Signal(list):
  def__call__(self,^∗^args,^∗∗^kwargs):
    for func in self:
      func(^∗^args,^∗∗^kwargs)
# usage example
def do_something(message):
  print message
sig = Signal()
sig.append(do_something)
sig(“hello world”)

The above class is not to be mistaken with the Qt signals that are used to connect to events originating from user input. The rationale behind using two different signal types is simple: the SamuROIData class was designed such that it is independent of any user frontend, and hence must not have a dependency on Qt.

This document view based design pattern permits the desired synchronized cooperation between the GUI and the Jupyter Notebook: as can be seen in **Figure 1A**, the Jupyter Notebook permits tuning of the GUI as well as data mutation and extraction via the SamuROIData class at the document level. Further, if user interaction with the GUI updates the SamuROIData class the same signal cascade as described above will be triggered.

To give an example of signal slot communication, we would like to describe in detail what happens during ROI mask addition. When a user adds a ROI mask to the data using the GUI widget, the widget’s Qt signal is triggered, which adds the ROI mask to the SamuROIData class, triggers its internal signal and notifies all interested listeners (for example the widget which displays the list of masks as a tree structure; upper part of **Figure 1B**). The widget, which adds the ROI mask, therefore, does not need to know about other components which also require notification: the logic is confined in the signals from the SamuRoiData object.

Because of this signaling structure, updates originating from the interactive shell will invoke the same mechanism and update relevant GUI elements (lower part of **Figure 1B**). On the other hand, one can also use the interactive shell to add custom GUI elements to an existing window, or connect post-processing and export functions to the data (see example in the online documentation). Another advantage of the separation between data and view is the future possibility to reuse the GUI code, e.g., in a cloud computing scenario. Then the data object behind the client GUI would simply defer all calculations and memory limitations to a server and present only 2D slices and the calculated traces of the data to the GUI.

#### Performance Considerations

For a smooth user experience and fast calculation of traces from the defined ROIs SamuROI always holds the full 3D video array in memory. With the use of double precision floating points and an assumed video size of 512 × 512 pixels and 1000 frames this results in about 2 GB of required RAM. Hence, long-term recordings with high frame rates are likely to exceed an average workstations system memory. Since features as memory mapped files are not supported in SamuROI, such datasets need to be split to fit into memory.

Calculating the time series of ROIs makes extensive use of numpy routines and has negligible CPU cost: due to numpy’s underlying C implementation a decent machine needs only a couple of milliseconds per ROI. Further, calculated traces get cached by SamuROI and hence need not be calculated twice. The only relevant computation times arise from the pre-processing of data (stabilization, filtering and/or renormalization) which can grow up to a couple of minutes per dataset. However, because pre-processing is usually run from an interactive python shell, it can easily be done in batch mode or distributed to dedicated machines. Then, the saved pre-processed data can be loaded into the GUI with minimal delay.

## Functionality and Results

We will now illustrate the functionality of SamuROI by describing the general workflow of data processing. After explaining data import and pre-processing, we describe the different widgets of the GUI and explain data export. We then provide three application cases at the micro-, meso- and macro-scale. We provide the most detailed description of micro-scale imaging, as we are not aware of any standardized freeware software solutions facilitating the analysis of fluorescence changes in complex dendritic structures.

### General Workflow

The first step of any image analysis software is the conversion of the acquired raw data into a format compatible with the analysis software. Depending on the data acquisition system used, dynamic image series are saved in a variety of data formats. We therefore needed to define a format that works with SamuROI. As an interface with SamuROI, we chose multiple image tif files. When it becomes necessary to convert data from other time series formats into multiple image tif files, we recommend the use of Fiji (Schindelin et al., 2012).

The first step after loading the multiple image tif file into SamuROI is the conversion into a 3D numpy array. This is a convenient format that allows a whole range of computations to be applied to the data. Usually, a couple of pre-processing steps are applied to the raw fluorescence images. Pre-processing can be performed in Python on this numpy array. SamuROI comes with a set of standard pre-processing functions. These include image stabilization [via opencv (Bradski, 2000), stabilization consists of rigid and warpaffine transformations to align each image to a given reference frame of the video provided], background subtraction, bandstop filtering and transformation of the raw fluorescence data into a ΔF/F dataset (see Supplementary Methods for details on the underlying algorithms). Usage of these functions requires the use of an interactive shell like Jupyter. With a basic working knowledge of Python, users can also implement their own custom pre-processing routines for the 3D numpy array.

Next, a SamuRoiData object is created from the pre-processed data. The SamuRoiData object can be visualized with its associated GUI. This data object can be accessed and manipulated from within this GUI or directly using python commands in the interactive shell or with stand-alone python scripts. The current version of the GUI can be used for ROI mask generation, smoothing, detrending, and thresholding.

We would now like to provide an overview of the current SamuROI GUI with all functional widgets. Our example data displays a dendritic segment with adjacent spines in a layer 2 cell of the MEC. The cell was in whole-cell patch clamp mode, the fluorescent Ca^2+^ signal corresponds to a doublet of backpropagating action potentials evoked by current injection.

**FIGURE 2:**
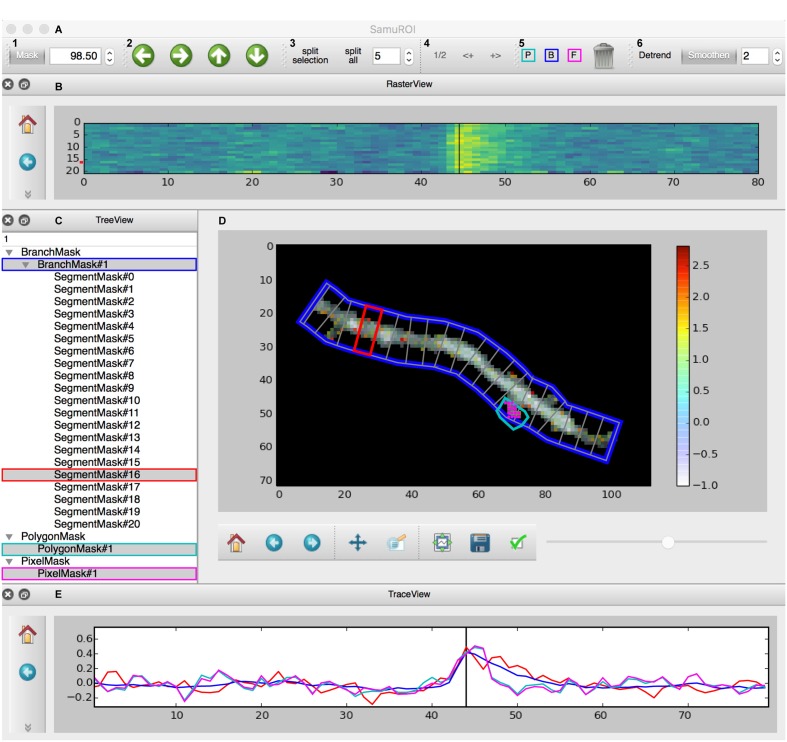
Screenshot of the GUI. **(A)** Toolbar: (A1) Allows the user to set a numeric cut-off value for the thresholding overlay mask. The thresholding overlay mask defines irrelevant background fluorescence and spares relevant pixels in the morphology image. (A2) Permits pixel-wise shifting of selected ROI masks in the direction of the arrows. (A3) Defines the width of branch sub-segments arranged perpendicular to the all BranchMasks (split all) or only the selected BranchMask (split selection). (A4) Permits grouping adjacent segments or splitting the selected segments in half. In tab (A5), the different mask drawing tools for PixelMasks (P, turquoise), BranchMasks (B, blue) and freehand PolygonMasks (F, magenta) can be selected. Selected masks can be deleted using the trashcan icon in this tab. Tab (A6) hosts the Detrend and Smoothen postprocessors. The degree of smoothing (moving average filter) can be defined using the number tab. **(B)** The RasterView widget visualizes the temporal and spatial distribution of the fluorescent Ca^2+^ signal over the segments of a selected BranchMask. Points on the *y*-axis corresponds to the segment number, the *x*-axis defines the frame number. The amplitude of the averaged fluorescent signal of a given segment is color-coded. The vertical black line between frames 40 and 50 corresponds to the slider button and the currently selected frame in **(D)** and the line in the TraceView **(E)**. The panel on the left gives access to standard matplotlib functions like for example zooming, panning or exporting of subplots as image files. Similar panels can be found in the FrameView **(D)** and TraceView **(E)** widgets. **(C)** The TreeView widget gives access to the ROI mask list. Different types of masks are grouped and selected masks are highlighted in gray. Names of the different ROI masks are indexed by default, but can be changed directly in the TreeView widget. **(D)** The image in the FrameView widget is a composite of the grayscale image of the morphology object with the thresholding overlay mask and the color-coded pixel brightness of the currently selected frame. The x- and y-scale corresponds to single pixels. ROI masks are projected on this image in light gray, selected ROI masks are highlighted in colors corresponding to traces demonstrated in **(E)**. The corresponding color code in **(C)** and (A5) is just for illustration. The scale bar on the right illustrates the color code for frame-specific pixel brightness. **(E)** In the TraceView widget, the relative change in brightness (in our example, the ΔF/F value) of the selected ROI mask(s) is plotted against the frame number.

The GUI is built using the PyQt library and consists of four interactive widgets and a toolbar (**Figures 2A–E**). The central ImageView panel (**Figure 2D**) displays a morphological grayscale image of the structure underlying the dynamic image series. A thresholding overlay mask defines the relevant pixels of the morphology image, which are above a user-defined threshold (see Supplementary Methods for details on the underlying algorithm). On top of the composite morphological grayscale and thresholding overlay mask image, a heatmap encodes the frame-specific fluorescence detected in each pixel. The threshold for the thresholding overlay mask can be set manually in the mask tab in the toolbar (**Figure 2A**) and a slider permits the user to explore the frame-specific fluorescence detected in each pixel frame by frame. After loading the dynamic image series into the GUI, the user can define specific ROI masks for further analysis of location-specific changes of fluorescence over time. The SamuROI toolbar supports creation of four types of ROI masks: branches, polygons, circles and pixel groups. Further, predefined segmentations [e.g., ROI masks exported from ilastik (Sommer et al., 2011) or swc files denoting dendritic structures from Neutube (Feng et al., 2015)] can be loaded via the interactive shell. The TreeView widget lists individually created or imported ROI masks (**Figure 2C**). While TreeView automatically generates names for individual ROI masks, the user can change names interactively. Selecting an item from the list in TreeView will display the corresponding trace of averaged intensity per frame in the TraceView widget (**Figure 2E**). Individual or all branch masks can be further subdivided into pixel-sized sub-segments using the ‘split’ tabs in the toolbar (**Figure 2A**). Individual sub-segments can be selected as children of individual branchmasks in the TreeView widget. The RasterView widget displays individual segments. The relative fluorescence of each segment is color coded and plotted against frame number (**Figure 2B**).

Within the GUI, SamuROI offers different post-processors like detrending and smoothing tabs or a pull-down menu item for event detection. Examples for how the interactive Jupyter shell can be used for additional post-processing of SamuRoiData objects are provided in the online documentation.

After defining and curating the ROIs and performing the necessary post-processing steps, the user needs to save the data. It is possible to document the analysis by saving the Jupyter Notebooks underlying individual experiments. In addition, we provide the option to export most of the relevant data stored in the SamuRoiData to hdf5 files. A pull-down menu in the GUI can be used directly to save the set of variables that is to be exported. At the moment this includes the threshold used to construct the thresholding overlay mask, ROI location and identity with the corresponding calcium imaging traces and the original 3D numpy dataset. User-specific post-processing results like those related to event detection can be incorporated into the hdf5 file, but this must be done outside of the GUI in Python. The hdf5 file is modeled on the structure of the SamuRoiData object, structured according to the masklist displayed in the TreeView widget. The analysis environment can be reconstructed from stored hdf5 files, which can be loaded into SamuROI as SamuRoiData objects. The hdf5 file structure allows the user to selectively import parts of a previous analysis environment, which makes it possible to easily reapply stored sets of ROI masks to a new dataset.

One key motivation for using the hdf5 file structure is that in large datasets, it often becomes necessary to identify individual events in different segments using automated procedures. Here, we define an event as form of electrical neuronal activity (an action potential or a synaptic response or a combination of both) that results in a temporary brightness change of the fluorescence indicator that can be clearly differentiated from baseline noise. Usually, events occur in different spatially confined segments of the data (e.g., different cell bodies at the meso-scale). Analyzed data, exported as hdf5 files, can be used for automated batch analysis in Python or other analysis environments. Batch processing of large datasets should best be performed on hdf5 files of individual experiments exported from SamuROI. However, for definition of the settings used for event detection and quality control, the SamuROI GUI is built to facilitate visualization of event detection. As a starting point, SamuROI offers standard, built-in event detection functionality based on template matching of a bi-exponential function. Briefly, this approach is based on defining a template of a typical event signal. This template then slides along the fluorescence trace and is scaled to fit the data at each point. This way, a point-by-point detection criterion is generated based on the optimal scaling factor and the quality of the fit. The user has to define the threshold above which the detection criterion defines an event (Clements and Bekkers, 1997). While originally developed for analysing electrophysiological data, this approach can also be applied in imaging applications (Tantirigama et al., 2017). Time constants, which define the fit parameters of representative ‘bait’ traces, must be obtained from other software solutions; we recommend the use of Stimfit (Schmidt-Hieber, 2014). Importing traces from hdf5 into Stimfit is relatively straightforward, which can then be used for curve fitting. Detected events are highlighted in the Treeview, Rasterview and TraceView widgets. Once the event detection settings (in our case time constants and detection criterion) have been optimized in the GUI, they can be performed in the Jupyter Notebook on larger datasets.

### Application Cases

#### Subcellular Imaging

**FIGURE 3:**
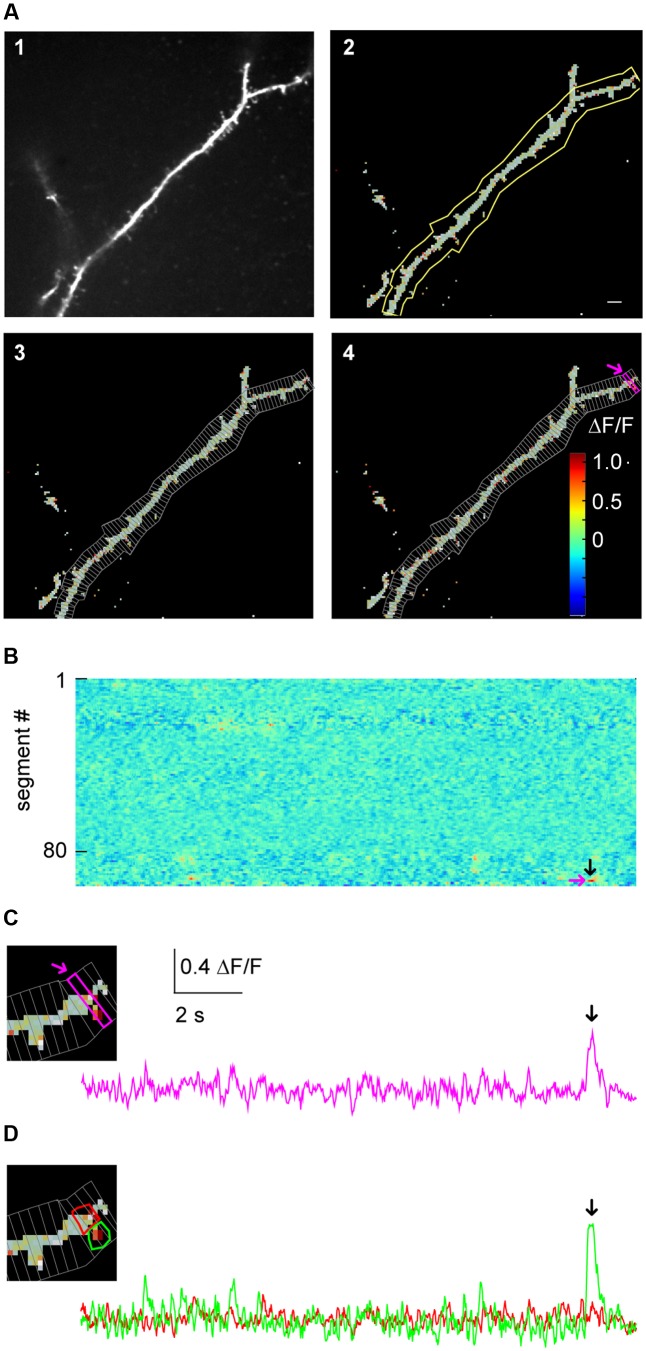
Micro-scale imaging example for using SamuROI on a dendritic structure. **(A)** (A1) The morphological image of a dendritic segment from an electroporated layer 2 cell in the MEC. (A2) The corresponding FrameView image from SamuROI, a composite of the thresholding overlay mask defining relevant pixels of the grayscale morphological image and color-coded frame specific brightness values of the relevant pixels. The yellow outline was generated using the BranchMask tool. The scale bar corresponds to 2 μm. (A3) Depicts how the BranchMask from (A2) is divided into SegmentMasks by SamuROI. **(B)** RasterView of segments from the dendritic branch corresponding to **(A)**. Time on the *x*-axis is defined by the scale bar in **(C)**. ΔF/F is color-coded as in (A4). The magenta arrow corresponds to the point on the *y*-axis representing the magenta-colored segment both in (A4) and in **(C)**. The black arrow corresponds to the time point indicated by arrows in **(C)** and **(D)** and defines the frame depicted in (A4). **(C)** The inset on the left magnifies the distal part of the dendrite shown in **(A)**. The trace corresponds to the SegmentMask outlined in the inset and (A4). **(D)** Based on the distribution of pixel brightness at the time point defined by the black arrow in **(B)**, ROI masks are defined manually as freehand PolygonMasks. The local Ca^2+^ transient in the spine (green) can be differentiated from the dendritic segment (red).

One intended use for SamuROI is the generation and visualization of temporal and spatial profiles of neuronal activity related Ca^2+^ signals from complex dendritic structures and spines (**Figure 3**). Specifically, this includes analysis of the spatial distribution of spontaneous synaptic events reflected by Ca^2+^ ‘hotspots’ on dendrites or spines. See Jia et al. (2010), Kleindienst et al. (2011) or Takahashi et al. (2012) for example research questions requiring this analysis approach. Another example is the identification and demarcation of spontaneous release from intracellular stores in dendrites. Related research questions can be found in Larkum et al. (2003), Miyazaki and Ross (2013) and Lee et al. (2016).

In our example, we would like to illustrate how SamuROI can be used to visually identify and localize a spontaneously occurring Ca^2+^ transient or ‘hotspot’ in a single spine on a long dendritic segment. Here, a layer 2 cell in the MEC has been electroporated with the Ca^2+^ indicator OGB1. A morphological image was generated as a maximum projection along time of the motion- corrected 3D dataset. Then, the motion- corrected 3D data set is transformed into ΔF/F data. SamuROI then transfers both, the morphology image and the ΔF/F 3D numpy array, into a SamuRoiData object. In the online supplements, we provide a Jupyter Notebook that includes a step-by-step description of data import, the pre-processing steps and the generation of the SamuRoiData object and the corresponding GUI.

Manual drawing of ROIs delineating subcellular structures like dendrites and spines requires the investigator to manually trace the boundary between the structure and the background, which is a time-consuming and tedious task. In SamuROI, we implemented a functionality that speeds this process up significantly. Based on the morphology image, SamuROI generates a ‘thresholding overlay mask.’ The software implements a thresholding algorithm (see Supplementary Methods) that defines above background pixels incorporated into the further analysis. Compare the raw morphological image **Figure 3A1** (top left) and the thresholded image **Figure 3A2** (top right). SamuROI ignores the masked out black pixels in ROIs, which only contain background fluorescence. ROI masks, irrespective of whether they are generated as tubes using the SamuROI branch tool or incorporated from somewhere else, can therefore be larger than the structure of interest. This speeds up manual ROI generation significantly and also facilitates the import of ROI masks from for example swc files, as ROI masks can include regions where no pixels are analyzed. Small inter-experimental changes in ROI shape are automatically incorporated, so that the same ROI can be used for consecutive sweeps of the same structure. Using of the same ROI mask for consecutive sweeps is further facilitated by an alignment tab (**Figure 2A2**) that permits shifting of selected ROIs. Together with the example Jupyter Notebook in the online supplements we also offer an example swc file from the freeware software Neutube together with an instruction how to generate swcs in Neutube that can be used by SamuROI and incorporated directly as branch ROIs.

The key objective of our application example is the detection of spontaneously occurring local hotspots of activity on a large dendritic structure. For this task, it is necessary to subdivide dendritic branches into segments and visualize fluorescence changes in each individual segment. Again, manual ROI drawing tools that are typically implemented in analysis software would now require the user to manually draw a large number of evenly spaced ROIs. For this task, it is necessary to subdivide dendritic branches into segments and visualize fluorescence changes in each individual segment. In SamuROI, the split tool (**Figure 2A3**) automatically divides tubular branchmasks into identically spaced sub-segments oriented perpendicular to the longitudinal axis of the dendritic branch (**Figures 3A3**,**4**). The spacing of these segments is a user-defined number of pixels. This adaptability is important, as signal to noise improves when the number of pixels in a segment corresponds to the number of pixels active in a hotspot. In each segment, the thresholding overlay mask defines pixels that will be averaged. Further, a pixel’s surface fraction, which resides within the ROI mask, determines its weight. Pixels in the interior of the ROI will have a weight of 1, boundary pixels will have a weight of less than 1.

**FIGURE 4:**
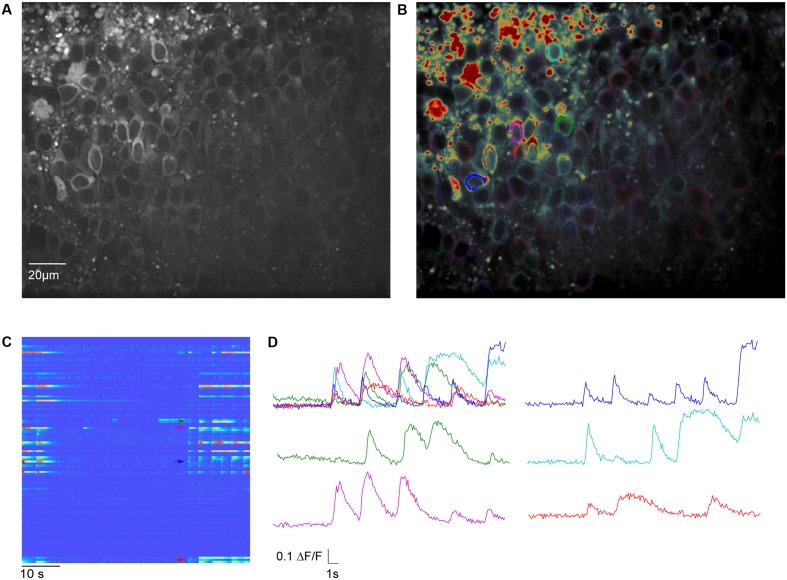
Meso-scale imaging example for using SamuROI neuronal populations in the piriform cortex of a brain slice. **(A)** 40× magnification GCaMP6f-fluorescence summed image of a horizontal slice from the AI95/NexCre mouse in layer 2 of the piriform cortex. **(B)** The summed image loaded into SamuROI in FrameView with an overlay of color-coded cellular ROIs obtained with our mask generating function. **(C)** The RasterView displays all ROIs that are plotted. It depicts the temporal and spatial profile of neuronal activity induced changes in GCaMP6f fluorescence during a spontaneous synchronous network event. The colored arrows correspond to the highlighted cells in **(B)**. **(D)** Fluorescence traces corresponding to segments color-coded in **(B)** and **(C)**. Arrows in **(C)** depict the starting points of the corresponding traces.

After SamuROI has calculated the average for all segments, it is necessary for the experimenter to identify and localize hotspots of activity. The usual output of ROI-Data are fluorescence traces. Visually screening large numbers of fluorescence traces derived from individual dendritic segments is tedious and prohibits immediate recognition of temporal and spatial patterns. Therefore, the RasterView widget (**Figure 3B**) provides a linescan-based color-coded display of the intensity time-course of each segment in a branch.

This approach enables the investigator to rapidly visualize the spatial and temporal activity pattern and identify a hotspot of activity in our example. In addition, once the putative hotspot has been identified in the RasterView widget, we want to know the exact position in on our morphological image in the frame view widget and visualize the underlying fluorescence trace to evaluate qualitative parameters of the signal which get lost in a heatmap. SamuROI offers a solution to this problem that imaging signals need to be displayed in different formats synchronously for evaluation. Our software permits intuitive browsing of the data by synchronizing different widgets in the GUI: in our example, clicking a temporally and spatially defined hotspot in the RasterView widget (arrows in **Figure 3B**) highlights the corresponding ROI mask in the FrameView widget (**Figure 3A4**) and the segment in TreeView. It also triggers the display of the corresponding sweep in TraceView (**Figure 3C**). We are now able to locate the signal at the distal tip of the dendritic segment and estimate the time course and the signal to noise ratio looking at the trace.

By definition, our segment masks are stereotyped and may not capture the perimeter of a hotspot or spine correctly. SamuROI enables the visualization of the exact spatial extent of the hotspot we detected in our example. By selecting the time points of interest in the RasterView widget at the event peak, the ΔF/F color-coded pixels are overlaid on the morphological image in FrameView (see inset in **Figure 3C**) and the corresponding frame is marked in the TraceView widget (**Figure 3C**, black arrow).

It is now necessary to define the hotspot in greater detail. For this purpose, we generated tools for ROI definition using freehand drawn polygons or individually selected pixel groups. In our example, the RasterView permits immediate identification of a hotspot and its localization in the FrameView widget. The intensity color-code in the FrameView widget demonstrates that the active pixels correspond to a dendritic spine (see the inset in **Figure 3C**). By drawing a freehand polygon around primarily active pixels in the FrameView widget, we manually generate a ROI mask that only incorporates the isolated hotspot (**Figure 3D**).

Using this example that illustrates the core functionality of SamuROI, we would now like to explain the options for further data processing offered by our hdf5 file based data format. From identification of a hotspot, one could save the adapted set of ROI masks (branch masks, segments and the newly generated polygon) and apply it to a different image series from the same structure. This way, it would be possible to identify and analyze all hotspots in a set of image series from the same structure. Additionally, one could use the detected signal as a ‘bait’ to generate a template for a typical signal and use this for automated event detection in this dataset. Once all image series are analyzed, the hdf5 files will not only contain all traces underlying labeled structures but also the spatial information related to these structures, which will be helpful when analysing spatial aspects of activity. One could for example analyze if hotspots tend to be spatially clustered or if they are distributed randomly.

#### Meso-scale Imaging

One of the goals of population imaging is to identify and describe structure in the activity of populations of cells. Specifically, single-cell Ca^2+^ signals representing action potential firing can be spatially and temporally related to each other during spontaneous network activity as for example in Namiki et al. (2013), or following extracellular synaptic stimulation as in Johenning and Holthoff (2007). *In vivo*, these cellular activity patterns are often related to behavior, one of many examples can be seen in Heys et al. (2014).

It is common in this kind of data exploration to have no hypothesis regarding where activity will be located or how it will be temporally structured within a population of cells. For this type of analysis the SamuROI GUI can be used for data visualization with generic ROI masks from a variety of software for interactive display of different groups of cells. In addition, the SamuROI GUI offers convenient functionality for manual curation of ROIs and for the testing of event detection parameters.

We would now like to give a specific example to highlight unique functionalities of SamuROI. In our example, we imaged immature spontaneous synchronized network events in a neonatal slice preparation of the olfactory cortex. In these network events, there is high synchrony between a subset of cells, which are hard to identify as single cells by established variance-based measurements relying on sparse firing (Hjorth et al., 2015; see discussion for details). Here, we present a workflow for measuring activity in densely packed cell populations that fire synchronously. We also show how our workflow can be used to provide a read out of the number of cells that are silent for the total duration of the recording.

For Ca^2+^ indication, constitutive GCaMP6f expression in post-mitotic excitatory neurons is achieved in the AI95/NexCre mouse line. The first step is to generate sets of ring-shaped ROI masks for GCaMP expressing cells that are based on pixel classification segmentation using ilastik (Sommer et al., 2011) and watershed segmentation using the scikit-image module in Python (van der Walt et al., 2014). In GCaMP-based datasets, the main underlying morphological feature is the ring shape of GCaMP6f expression, with a fluorescent cytosolic rim and a dark central nucleus (**Figure 4A**), and we present a segmentation approach specified for this morphological pattern. The generated ROIs are illustrated in **Figure 4B**.

In the online supplements, we provide an example Jupyter Notebook for using these functions to generate single cell segmentation ROI masks and opening them in SamuROI. In the documentation we also outline how to generate ROI masks using ilastik. After automated ROI generation in ilastik, we implemented a manual correction step for adding and deleting single cells. The user input required is essentially a mouse click on the dark nuclear center of a ring shaped cell. The final outcome of our segmentation is a 2 dimensional array in which each cell is denoted by a different number (i.e., every pixel belonging to cell 1 is denoted by a 1 in the image). This array is then imported into the SamuROI GUI. SamuROI works with these segmentations and treats them just as though they were a set of individual ROIs.

Basic GUI functionality of meso-scale population ROI masks is similar to that described above for micro-scale data. The GUI displays the mean fluorescence of all pixels in each mask and displays this through RasterView and TraceView as can be seen in **Figures 4C,D**. RasterView reveals structured activity in cell populations and allows event selection that leads to highlighting of the cell of origin in both FrameView and TreeView, as well as plotting in TraceView. Additionally, single or multiple cells can be selected in FrameView for simultaneous viewing and comparison of activity in TraceView, which is shown in **Figure 4C**. This way, it is possible to intuitively visualize aspects like synchrony, number of cells participating and the order of neuronal activation during events. One can pick cells displaying different activity patterns in the RasterView (e.g., the blue cell showing a large number of small bursts and the green cell showing a small number of large bursts), directly visualize their location in FrameView and compare the underlying traces in TraceView. In addition, it is possible to add more ROIs using the GUI. An example how this could be used in an experiment to bridge subcellular micro- and meso-scale imaging would be simultaneous imaging of a meso-scale population and single dendritic branches of individual dye-filled cells. This example would require the addition of branch segments to the cell ROI masks, which can be easily accomplished in SamuROI.

The SamuROI GUI further permits standard post-processing and event detection functionality of population imaging data sets. Data export as hdf5 files currently needs user intervention from the Jupyter Notebook, as the standard pull down menu does not offer the export of cell-specific ROI masks. In our online Supplementary Material, we provide a short function that enables SamuROI to add cell-specific ROI masks to the hdf5 files.

Representative traces visualized in the GUI can be picked and exported to other software, such as Stimfit to generate curve templates that permit automated event detection. The GUI can then be used to test sensitivity and specificity of event detection parameters in individual experiments before batch processing the hdf5 files in Python directly. This can be done using the same functions that have been used in the GUI. Batch analyzed data will provide spatial and temporal information of detected events in the hdf5 files, which will enable the user to extract spatial and temporal correlations of network activity simultaneously.

#### Macro-scale Imaging

Low magnification imaging of brain-activity induced changes in Ca^2+^ indicator fluorescence (or, in principle any other indicator of neuronal activity employing changes in brightness as a readout) enables researchers to analyze the spatiotemporal spread of activity patterns over different brain regions with low spatial and high temporal resolution. Specific uses of macro-scale imaging include the spatial and temporal spread of spontaneous activity in brain slices (Easton et al., 2014) or interregional synchrony *in vivo* (Busche et al., 2015).

**FIGURE 5:**
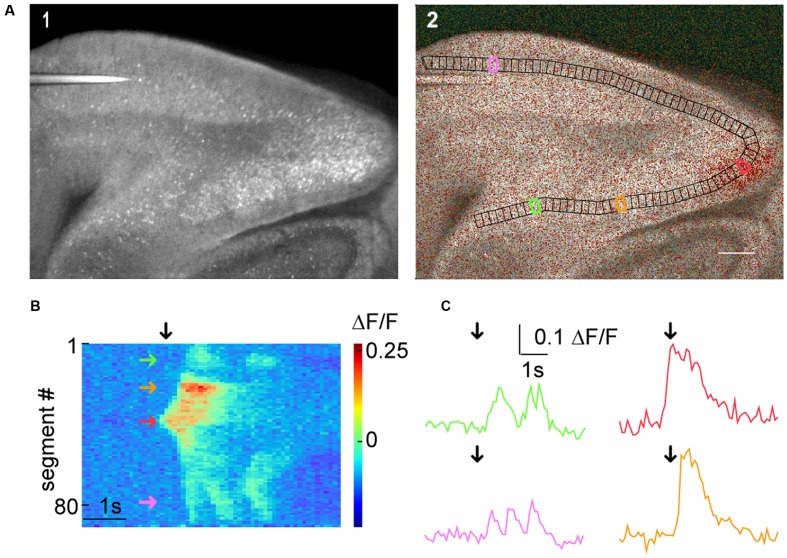
Macro-scale imaging example for using SamuROI on different cortical regions of a brain slice. **(A)** (A1) A low magnification GCaMP6f-fluorescence image of a sagittal slice from a AI95/NexCre mouse. Different parts of the hippocampal formation are visible. (A2) Shows a morphological image with an overlay of color-coded frame specific brightness value. In black, we see a BranchMask over several hippocampal regions (subiculum, presubiculum, parasubiculum, medial entorhinal cortex, and lateral entorhinal cortex) divided into equally spaced segments. The scale bar corresponds to 200 μm. **(B)** The RasterView corresponds to the segmented BranchMask (A2). It depicts the temporal and spatial profile of neuronal activity induced changes in GCaMP6f fluorescence during a spontaneous synchronous network event. The green arrow points to a segment in the subiculum, the orange arrow to the presubiculum, the red arrow to the parasubiculum and the magenta arrow to the lateral entorhinal cortex [also see segments highlighted in corresponding colors in (A2)]. The black arrow defines the point on the *x*-axis that corresponds to the frame shown in (A2) and the time points depicted by black arrows in the traces in **(C)**. **(C)** Fluorescence traces corresponding to segments color-coded in (A2) and **(B)**.

The generic functions of SamuROI can be used to facilitate interpretation of macro-scale datasets. In our example, we would like to demonstrate how the spatiotemporal structure of a spontaneous synchronized network event is intuitively visualized and related to different brain structures using SamuROI. GCaMP6f is expressed using the AI95/NexCre mouse line. **Figure 5A** displays a sagittal slice of the parahippocampal formation, where neonatal spontaneous synchronized network events were imaged. A question we want to answer using SamuROI in this example is how the horizontal (lateral) spread of the signal in superficial layers of the parahippocampal formation is organized in time and space. The branch ROI tool we initially developed for micro-scale imaging is especially well suited for this task, demonstrating how SamuROI can be applied for image analysis flexibly at different spatial scales. As branch ROIs can have any user-defined width and direction, it is possible to generate a ROI incorporating the adjacent brain regions subiculum, presubiculum, parasubiculum and entorhinal cortex (**Figure 5A2**). The incorporation of deep and superficial layers can be adjusted by modifying the width of the branch ROI mask. Using the segmentation tool, we then divide these cortical regions into sub-regions at arbitrary spatial resolution (**Figure 5A2**). A RasterView of the sub-regions then displays the temporal and spatial dynamics of neuronal activity reflected by changes in fluorescence (**Figure 5B**) and the user can then localize individual signaling patterns like the leading edge of a wave (**Figure 5B**, red arrow) or an oscillating structure (**Figure 5B**, green and purple arrow). After clicking on the corresponding part of the RasterView widget, the corresponding segment is localized in the FrameView widget (**Figure 5A2**). The TraceView widget displays the corresponding traces (**Figure 5C**). Based on the different spatiotemporal patterns extracted from the RasterView, it is possible to draw freehand polygon-ROIs based on different patterns. This is facilitated by the time-locked intensity color code in the FrameView widget. In our example, this highlights the initiation of the signal in the parasubiculum.

## Discussion

When studying neuronal activity with imaging, the appropriate analytical unit depends on the scientific question and size scale. Depending on spatial resolution, these analytical units could be, for example, dendritic branches, spines, single cell bodies or cortical layers and they ideally represent a unit of neuronal or network computation. Researchers aim to extract fluorescence changes specific to these analytical units, based on which they visualize, detect and localize neuronal activity patterns. Technological progress challenges researchers with the opportunity to generate increasingly complex datasets in which the ideal spatial scale is often hard to define or predict in advance.

SamuROI is built to meet the rising demand for analysis freeware. It provides an intuitive and convenient workflow for data exploration and ROI creation at arbitrary spatial scales. SamuROI is a Python-based, open source analysis environment for image series of intensity changes of fluorescent indicators over time. The software permits both data browsing and deep analysis using Python by seamlessly integrating command-line interactions with a user-friendly GUI, achieved by using Jupyter Notebooks.

As such, the software has several core strengths:

• Simplified identification of complex spatiotemporal patterns by human observation that would otherwise get lost in highly complex datasets.
• Time effective ROI management and manual curating of automatically generated ROIs from other software solutions.
• Instantaneous switching between temporal and spatial aspects of the data via interactive point and click widgets.
• Facilitation of quality control in terms of the fluorescent signal, ROI segmentation and event detection that is presented to the user.

The tool is straightforward to install. The online documentation includes code templates to illustrate usage and enable ‘out of the box’ use with Jupyter Notebook. While Jupyter is the recommended platform for running the GUI it is also possible to use SamuROI as a stand-alone application. The modularity of the pipeline permits each processing stage to be carried out independently, including pre-processing, data visualization, ROI definition, data export and event detection. Data are exported as hdf5 files, which contain all necessary information for further batch processing of the data. The package is carefully documented and open source to permit further collaborative development.

SamuROI is complementary to other existing imaging analysis software like Fiji (Schindelin et al., 2012) and SIMA (Kaifosh, 2014). These tools offer different data processing, visualization and exploration options than SamuROI. A unique feature of SamuROI is the document-view pattern based framework that permits online modification of objects in the SamuROI GUI in Python using an interactive shell like Jupyter and vice versa.

An example of integration of Fiji and SamuROI is the excellent file conversion functionality of Fiji, which enables the conversion of a larger number of file formats into Multi-tif files that can be read out by SamuROI. While Fiji offers both a neurite tracer and a ROI manager for fluorescent time series, to our knowledge there is no default way of combining the two. We found the visualization and manual curating options of ROIs generated with the Fiji ROI manager limited as there are no point and click widgets. These tools offer different visualization and exploration options to SamuROI, and can be easily used in parallel. While SIMA focuses on meso-scale population Ca^2+^ imaging *in vivo*, SamuROI aims to provide an integrated analysis environment for imaging data at what we define as the micro-scale, meso-scale and macro-scale. In addition, SIMA offers the ROI Buddy, an excellent segmentation tool for manual curating of ROIs. However, we missed an intuitive display that permits visualization and browsing of fluorescence traces. However, SamuROI by no means aims to replace any of those tools, and we encourage using these tools in parallel. For example, one might prefer to use the frame alignment procedures and ROI Buddy segmentation in SIMA as a pre-processing step followed by further analysis and visualization/exploration of the data in SamuROI. This would be an easy way to incorporate activity-based pixel correlations (Junek et al., 2009; Mukamel et al., 2009; Tomek et al., 2013; Kaifosh, 2014; Hjorth et al., 2015; Pnevmatikakis et al., 2016) to the analytical pipeline and these can be further edited in SamuROI.

One of the most critical and, when performed manually, time-consuming steps of dynamic image series analysis is the definition of ROI masks. For micro-scale Ca^2+^ imaging, we are not aware of an integrated software solution that permits both semi-automatic ROI mask generation and data browsing/analysis. On the other hand, for semiautomatic tracing of morphological data, many freeware software tools are already available for morphological segmentation of images. Software solutions like Neutube (Feng et al., 2015), Neuronstudio (Wearne et al., 2005; Rodriguez et al., 2008) or the simple neurite tracer plugin for Fiji (Longair et al., 2011) permit semi-automatic tracing of dendritic and axonal structures. SamuROI is built to interact with these, as any ROI pattern can easily be converted into an array of pixels that can be added to the attribute masks. In our online supplement, we provide examples that illustrate how ROI sets compatible with SamuROI can be generated from freeware programs validated for structure recognition. SamuROI can read SWC files (e.g., exported using Neutube (Feng et al., 2015)) and flatten these 3D dendritic tree structures into 2D branch masks. This greatly facilitates the generation of branch specific ROIs, and provides a good example how the excellent branch tracing functionality of Neutube can be combined with SamuROI.

In contrast to micro-scale imaging, there are many tools facilitating the detection of cell bodies in population Ca^2+^ imaging on the meso-scale. A number of recently developed approaches define pixels belonging to active cells based on variance in brightness using activity-based pixel correlations (Junek et al., 2009; Mukamel et al., 2009; Tomek et al., 2013; Kaifosh, 2014; Hjorth et al., 2015; Pnevmatikakis et al., 2016). These variance-based approaches work well for identifying sparsely active cells, but cannot detect silent cells nor can they always distinguish between closely packed synchronously active cells that do not fulfill the prerequisite of statistical independence. A recently published approach directly addresses this issue for postnatal early synchronous network activity (Hjorth et al., 2015). Regardless of the method used to detect cells, SamuROI can provide a useful environment for visualization and quality management of the resulting ROIs. We also provide example functions that implement polygon ROI mask creation for inactive and synchronous cells using the machine-learning based structure recognition software ilastik (Sommer et al., 2011), together with python functions based on scikit-learn and the standard python library.

### Outlook

SamuROI works well with existing tools and streamlines the analysis of dynamic image series such as those acquired using Ca^2+^ indicators. SamuROI has many built in features covering a complete pipeline of data processing and analysis. While many software packages for dynamic image series analysis exist, many necessary features missing from these packages have been combined into SamuROI. Since SamuROI permits the easy import of ROI masks generated (semi-) automatically with other software tools, we do not prioritize the implementation of new segmentation algorithms in future versions of the software. Our software has been designed in such a way that event detection algorithms different from the template based algorithms based on (Clements and Bekkers, 1997) can be easily implemented. SamuROI will be used as a versatile tool for data exploration and analysis, for identifying meaningful structure in complex datasets and for convenient ROI management. SamuROI together with sophisticated structure recognition software minimizes the need for human supervision in selecting pixel-defined structures of interest. This should allow scientists to focus their attention on data scanning for recognition of meaningful patterns in the data and quality control.

## Author Contributions

MR and SL wrote code. FJ, LM-V, DP, and SL contributed example data. FJ, MR, and SL were involved in conceptualizing the software. FJ, LM-V, DP, SL, MR, SR, and DS designed and tested the software. FJ, SL, MR, SR, and DS prepared figures and wrote the manuscript.

## Conflict of Interest Statement

The authors declare that the research was conducted in the absence of any commercial or financial relationships that could be construed as a potential conflict of interest.

## Footnotes

1 https://github.com/samuroi/SamuROI

2 https://samuroi.readthedocs.io

3 https://www.jax.org/strain/024105

4 https://github.com/samuroi/SamuROI

5 https://samuroi.readthedocs.io
