# Supplementary material for: SamuROI, a Python-Based Software Tool for Visualization and Analysis of Dynamic Time Series Imaging at Multiple Spatial Scales
Source: Front Neuroinform. 2017 Jun 29;11:44. doi: 10.3389/fninf.2017.00044 (PMC5489661; doi:10.3389/fninf.2017.00044)
Supplement: Supplementary file 1 [file Data_Sheet_1.docx]

**Supplementary Methods**

Overlay thresholding algorithm

SamuROI can use a two-dimensional grey scale morphology image to automatically create a mask around relevant objects in the morphology.

The mask can be visualised from the GUI and has to be validated manually. It is calculated as follows:

1. compute percentile of morphology image and use it as initial threshold value
2. apply Sobel filter (from scikit image) to morphology image and create an elevation map
3. create a marker image where pixels get marked that are close to the threshold
4. run watershed algorithm (from scikit morphology)
5. user inspects result and potentially adapts threshold value

For the specific implementation see the SamuROIData implementation and the scikit image documentation.

Fluorescence renormalization

SamuROI comes with three different ways to renormalize the raw fluorescence data F which depends on space (x,y) and time (t):

1. via standard deviation: in this case the video data is segmented into different blocks, each containing B frames. Then the standard deviation is calculated for every pixel in each block over the frames of that block. Then the block with the minimum standard deviation is selected for each pixel and the baseline fluorescence is calculated as the mean of these blocks resulting in a baseline image F_0_(x,y)
2. via a linear fit: To account for small bleaching effects a linear fit is calculated for the time series of each pixel. With the resulting slopes m_x,y_ and offsets o_x,y_, F_0_ is defined as F_0_(x,y,t)=m_x,y_t + o_x,y_.
3. via the median over individual frames: in this case F_0_ does not depend on spatial coordinates and is simply defined as F_0_(t)=median(F(x,y,t)), where the median operates on all pixels of individual frames.

In all cases the calculation of ∆F/F = (F-F_0_)/F_0_ from the above definition is implemented via numpy’s array broadcasting functionality.
